# Building a global policy agenda to prioritize preterm birth: A qualitative analysis on factors shaping global health policymaking

**DOI:** 10.12688/gatesopenres.13098.1

**Published:** 2020-06-22

**Authors:** Sara Kassabian, Sara Fewer, Gavin Yamey, Claire D. Brindis

**Affiliations:** 1 Institute of Global Health Sciences, University of California San Francisco, San Francisco, CA, USA; 2 Evidence to Policy Initiative, University of California San Francisco, San Francisco, CA, USA; 3 Duke Global Health Institute, Duke University, Durham, North Carolina, USA; 4 Philip R. Lee Institute for Health Policy Studies, University of California San Francisco, San Francisco, CA, USA

**Keywords:** preterm birth, newborn health, newborn survival, global health, health policy

## Abstract

**Background: **Preterm birth, defined as infants born before 37 weeks of gestation, is the largest contributor to child mortality. Despite new evidence highlighting the global burden of prematurity, policymakers have failed to adequately prioritize preterm birth despite the magnitude of its health impacts. Given current levels of political attention and investment, it is unlikely that the global community will be adequately mobilized to meet the 2012 *Born Too Soon* report goal of reducing the preterm birth rate by 50% by 2025.

**Methods**: This study adapts the Shiffman and Smith framework for political priority to examine four components contributing to policy action in global health: actor power, ideas, political context, and issue characteristics. We conducted key informant interviews with 18 experts in prematurity and reproductive, maternal, newborn, and child health (RMNCH) and reviewed key literature on preterm birth. We aimed to identify the factors that shape the global political priority of preterm birth and to describe policy opportunities to increase its priority moving forward.

**Results**: The global preterm birth community (academic researchers, multilateral organizations, government agencies, and civil society organizations) lacks evidence about the causes of and solutions to preterm birth; and country-level data quality is poor with gaps in the understanding required for implementing effective interventions. Limited funding compounds these challenges, creating divisions among experts on what policy actions to recommend. These factors contribute to the lack of priority and underrepresentation of preterm birth within the larger RMNCH agenda.

**Conclusion**: Increasing the political priority of prematurity is essential to reduce preventable newborn and child mortality, a key target of the 2030 Sustainable Development Goal for health (target 3.2). This study identifies three policy recommendations for the preterm birth community: address data and evidence gaps, clarify and invest in viable solutions, and bring visibility to prematurity within the larger RMNCH agendas.

## Introduction

Preterm birth, which refers to infants born before 37 weeks of gestation, is a significant contributor to child and newborn mortality worldwide. An estimated 14.8 million newborns were born premature in 2014, and in 2016, 18% of child deaths were attributed to complications of preterm birth, making prematurity the leading cause of death for children under five years of age ^1, 2^. Premature infants that do survive are more likely than infants born at term to suffer from a range of morbidities, including respiratory distress, sepsis, difficulty feeding, and cerebral palsy, among other conditions ^1^. Although preterm birth occurs across communities and geographies, 80% of preterm births occur in south Asia and sub-Saharan Africa ^1^.

In 2012, a seminal report, *Born Too Soon: The Global Action Report on Preterm Birth,* led by the March of Dimes, the Partnership for Maternal, Newborn & Child Health (PMNCH), Save the Children, and the World Health Organization (WHO), provided the first ever estimates of the global burden of preterm birth ^3^. It also introduced a global action agenda to reduce the preterm birth rate by 50% by 2025.

However, progress has been slow and preterm birth remains largely hidden on the global policy agenda. Reducing preterm birth is essential to achieve the Sustainable Development Goal (SDG) for health (SDG 3), including the key targets on newborn and child mortality, yet prematurity is not measured in the indicators or monitoring frameworks ^1^. Similarly, the *Every Woman Every Child Global Strategy for Women’s, Children’s and Adolescents’ Health* (2016–2030) has a strong focus on newborns and preventing stillbirths, but not prematurity ^4^. In addition, most countries do not report national data on preterm births and the World Bank’s Global Financing Facility for Women, Children, and Adolescents (GFF) does not monitor preterm birth rates ^5^.

In order to reduce preterm births, prematurity must gain increased political priority – that is, “the degree to which international and national political leaders actively give attention to an issue, and back up that attention with the provision of financial, technical, and human resources that are commensurate with the severity of the issue” ^6^.

This study examines the actors, ideas, political contexts, and characteristics that have shaped the visibility of preterm birth, and identifies opportunities and challenges for prematurity to gain greater political priority moving forward. We focus on the global level because preterm birth is a worldwide health challenge, not limited to a particular geography.

## Methods

### Ethics

The University of California San Francisco (UCSF) Committee on Human Research certified this study, IRB number 15-15752, as exempt. We obtained written, informed consent from all key informants. Informants were assigned a unique identifier (e.g., Key Informant 1, KI1) to maintain anonymity.

### Political priority framework

We applied a conceptual framework developed by Shiffman and Smith to assess the political priority of preterm birth ( Table 1) ^7^. According to this framework, a global health issue achieves political priority when: (1) international and national political leaders publicly and privately express sustained concern for the issue; (2) the organizations and political systems they lead enact policies to address the problem; and (3) these organizations and political systems provide resources to address the problem that are commensurate with its severity ^7^. The framework has been applied to several underrepresented health challenges, including safe motherhood, newborn survival, mental health, and surgical care to understand the factors contributing to or hindering policy action ^7– 10^. Shiffman and Smith propose that there are four key determinants of political priority in the context of a particular health issue: actor power, ideas, political context, and characteristics of the issue; and 11 sub-factors, summarized in Table 1 ^7^. The collective impact of these factors determines the degree of prioritization or neglect of a given health challenge, which in this instance is preterm birth ^7^.

**Table 1.** Adaptation of the Dare, AJ, *Prioritizing Surgical Care on National Health Agendas: A Qualitative Case Study of Papua New Guinea, Uganda, and Sierra Leone*.

Components	Description	Factors that shape political priority in Preterm Birth
Actor power	The strength of individuals and organizations concerned with preterm birth.	1. Political community cohesion: the level of connectivity among the network of individuals and organizations involved with preterm birth at a global level. 2. Leadership: Presence of individuals that can unite the community, and are recognized advocates for prematurity. 3. Guiding institutions: Effectiveness of organizations with a mandate to lead the initiative. 4. Grassroots advocacy: The level of mobilization among community leaders to advocate for preterm birth at the national and global level.
Ideas	The ways in which those involved with preterm birth understand and portray it.	5. Internal frame: The mutual agreement of the policy community on definitions of, causes of, and solutions to the problem of preterm birth. 6. External frame: Public representations of an issue that gains resonance with external audiences, and particularly political actors.
Political contexts	The environments in which actors operate.	7. Policy windows: Political moments where conditions align well for preterm birth, introducing an opportunity for advocates to influence decision makers. 8. Global governance structure: The norms and institutions operating in the sector offer a platform for united action.
Issue characteristics	The features of the problem of prematurity.	9. Credible indicators: Clear and measurable data is available that demonstrates the severity of preterm birth and allows for accessible monitoring. 10. Severity: The size of the burden of the health issue relative to other problems, such as mortality or morbidity levels. 11. Effective interventions: The extent to which interventions for preterm birth are cost-effective, evidence-based, and simple to implement.

Source: Adapted from: Dare AJ, Lee KC, Bleicher J, Elobu AE, Kamara TB, Liko O, et al. (2016) Prioritizing Surgical Care on National Health Agendas: A Qualitative Case Study of Papua New Guinea, Uganda, and Sierra Leone. PLoS Med 13(5): e1002023. doi: 10.1371/journal.pmed.1002023 ^9^ under the terms of the Creative Commons Attribution 4.0 International license (CC-BY 4.0).

### Data collection and analysis

We conducted 18 semi-structured interviews with key informants (KIs) from May 2015 to September 2015. We purposively selected KIs based on their expert knowledge of preterm birth as researchers, practitioners, advocates, and policymakers at the global level and in lower-income countries (LICs) and middle-income countries (MICs). We identified KIs by reviewing peer-reviewed and grey literature on preterm birth and on reproductive, maternal, newborn, and child health (RMNCH), consulting professional networks, and asking KIs for other relevant professionals (i.e., we combined purposive sampling with snowball sampling) ^11, 12^. SK contacted prospective KIs by email asking if they would be interested in participating in our study. If they expressed interest in participating in the study, we set up a time to connect by phone, Skype, or in-person using email correspondence. Our sample included basic scientists, epidemiologists, and representatives from major bilateral and multilateral organizations, academic research groups, advocacy groups, and the largest research funders in the preterm birth arena ^11^.

Interviews were conducted until our study reached theoretical saturation, i.e., no new themes were emerging in our interviews ^12^.

We used the Shiffman and Smith framework to develop the semi-structured interview guide, which informed the interviewing process and analyses (see *Extended Data* ^13^). Interviews were conducted in English in-person, by telephone, or on Skype, with the average interview lasting about one hour. Interviews were audio recorded and transcribed. The audio recordings from the interview were deleted after the de-identified transcripts were created. The de-identified transcripts are secured on a password protected and encrypted computer.

Interview transcripts were analyzed using Dedoose, version 7.1.3, a data management software for qualitative research ^14^. A codebook was developed based on emerging themes, which began with an open-ended “impressions coding” exercise and then was narrowed and refined by two researchers (SK and SF) (See *Extended Data* ^13^). Each interview was coded by two researchers SK and SF to ensure consistency ^15^. Each interview was summarized to identify key themes, as were codes across all interviews. This process was documented with internal memos and matrices to check bias and maintain transparency. All authors discussed the summary data to identify findings and ensure the interpretations were sound and replicable.

The interview data was supplemented with a review of published and grey English language literature on preterm birth from 2015–2019. SK searched PubMed and Google for the relevant literature. This literature was identified by searching for articles about preterm birth and political priority, using search terms such as “prematurity” or “preterm birth” and “advocacy”, “priority”, “politics”, and “leadership”. In addition to providing context and verification to key informant remarks, this literature allowed us to track policy changes for preterm birth from 2015–2019 to see whether the early years of the SDGs affected attention to the issue.

## Results

### Actor power

Our study found that the global preterm birth community consists of academic researchers, multilateral organizations, government agencies, and civil society organizations that are well connected in part because of a shared history of collaboration and group decision-making.

After publishing the *Born Too Soon* report in 2012, the report authors and supporting organizations collectively decided to expand the group’s focus from addressing preterm birth as a single cause of newborn death to addressing newborn survival more broadly (KI2-3, KI15) ^16, 17^. This expansion aligned with the report’s call for partners across the RMNCH continuum of care to work collaboratively to address preterm birth (KI1-4, KI6-7, KI10, KI15). In 2014, core partner institutions behind *Born Too Soon* joined child health institutions to create the Every Newborn Action Plan (ENAP) to end preventable newborn deaths and stillbirths by 2035 (KI 2-3, KI10, KI13, KI15). ENAP has become the main organizing body for preterm birth, within the larger newborn survival network.

An agreement of those partners at that moment was that we needed to broaden this action plan to include not just preterm birth, but other main causes of newborn death. (KI15)

Informants described a high degree of collaboration under the ENAP platform (KI1-5, KI11, KI13, KI15-18). A variety of institutions helped guide the ENAP’s strategy ( Table 2), with the WHO responsible for establishing norms and standards, as well as convening policymakers and practitioners (KI1-18). The ENAP engaged countries to reach newborn health milestones, and also spurred preterm birth initiatives, including the Public Private Partnership to Prevent Preterm Birth and Born on Time.

**Table 2.** Actors Guiding the Every Newborn Action Plan ^16^.

**Every Newborn Action Plan steering committee**	
• The Bill & Melinda Gates Foundation • London School of Hygiene & Tropical Medicine • Maternal Health Task Force • Aga Khan University • Maternal and Child Health Integrated Program (MCHIP) • The Partnership for Maternal, Newborn & Child Health (PMNCH) • Save the Children • United Nations Foundation • Global Alliance to Prevent Prematurity and Stillbirth (GAPPS)	• United Nations International Children's Emergency Fund (UNICEF) • United Nations Population Fund (UNFPA) • United States Agency for International Development (USAID) • Maternal and Child Survival program • World Health Organization
**Supporting partners**	
• American Academy of Pediatrics • Canada • Children’s Investment Fund Foundation • Core Group • Council of International Neonatal Nurses (COIN) • Development Media International • Elma Foundation • European Foundation for the Care of Healthy Infants (EFCNI) • Global System for Mobile Communications (GSMA) • International Confederation of Midwives • International Federation of Gynecology and Obstetrics (FIGO) • International Pediatric Association • Johns Hopkins Bloomberg School of Public Health	• Johnson & Johnson • Laerdal • Neonatal Alliance • Makerere University • March of Dimes • MDG Health Alliance • University of Pretoria • Norad • PATH • Peking University Center of Medical Genetics • Sick Kids, Centre for Global Child Health • SNV • UK aid • University College London (UCL)_ • White Ribbon Alliance • Women Deliver • World Vision

I think that one of the things that people would say about the newborn health community is that it's a very collaborative and quite a highly networked group. (KI3)

Informants highlighted the strength of the global newborn network’s technical capacity (KI1, KI3) ^18^. Academics have played a leadership role in advancing research on preterm birth and shaping the newborn survival community (KI5, KI8-10, KI18). Some informants noted that the prominent role of academics has created barriers to communicating with advocates and policymakers (KI2, KI10). In addition, some informants expressed concern that donors invested $100 million in a UCSF research initiative on preterm birth, as opposed to funding an institution such as the WHO (KI2-3, KI6).

A notable weakness within the preterm birth community was the lack of political and civil society champions from LICs and MICs. Whereas the March of Dimes has mobilized civil society on preterm birth in the United States, grassroots advocacy in LICs and MICs has been insufficient (KI1-2, KI3-6, KI10-12, KI14-15, KI17-18). Informants mentioned several barriers to civil society engagement in LICs and MICs, including stigma toward mothers of preterm infants and women’s disempowerment (KI1-3, KI9-10, KI12, KI18); lack of awareness of the problem of prematurity (KI12, KI17); perceptions of feebleness of the infant or death of premature infants as fate (KI3, KI11-12, KI16); and a lack of external development funding for preterm birth (KI10, KI14).

Frankly, to think of a grassroots movement would imply that there is knowledge of the nature of the problem. And, frankly, people are not aware that prematurity can be prevented. Again, the vast majority of the population has a sense of inevitability. You lose a baby [then] it was God's will. It was Mother Nature, it was not meant to be. And that is not true. (KI12)

### Ideas

This study found that a lack of evidence on the causes of and solutions to preterm birth has created divisions on how to prioritize prevention and care agendas, which in turn hinders how preterm birth is framed externally to policymakers.

There are some signs of cohesion within the preterm birth community. For instance, the field follows the WHO’s definition of preterm birth as any baby born before 37 completed weeks of gestation (KI1-16, KI18) ^3^. Many informants also described a shared understanding of the best care practices for preterm infants, such as essential newborn care and emergency services for mothers (KI1-7, KI9-10, KI13-15, KI17) ^16^.

However, informants described weak metrics on the effectiveness of existing interventions at the local level (KI2, KI4, KI11) and noted knowledge gaps on how to best implement effective care interventions in LICs and MICs (KI1-4, KI9, KI14-15). They called for a coordinated effort around discovery and implementation science (KI11, KI16).

Furthermore, there was disagreement on how to allocate constrained resources, namely between prevention and care strategies (KI1-2, KI4-11, KI15-16). Informants referenced a range of different prevention strategies, such as family planning, prenatal care, and nutrition interventions, as a means to address the range of risk factors associated with preterm birth. However, informants warned that because the underlying etiology of preterm birth is still largely unknown, the mechanisms for preterm birth prevention are complex and not well understood (KI2, KI8-10) ^19– 23^.

Informants agree there is an urgent need for more evidence on prematurity, but there was a lack of consensus as to what types of prevention research should be the first priority (KI1-2, KI4-12, KI15). Suggestions included scientific discovery for new prevention methods (KI4, 7, KI11-12, KI16) and improving the scale-up of existing prevention interventions, such as the safe administration of antenatal corticosteroids for mothers at risk of preterm delivery (KI2-3, KI5, KI10-11).

Informants reported that the limited evidence around preterm birth prevention has stalled progress in engaging policymakers on prevention strategies (KI1-2, KI4, KI8-9). While some KIs warned that the prevention agenda has been neglected too long, others were hesitant to promote interventions without more evidence (KI4, KI7, KI11-12). Two informants reported that the Public-Private Partnership to Prevent Preterm Birth encountered resistance because researchers felt it was presumptive to create a prevention initiative when there is a lack of scientific clarity (KI5, KI10).

When we started to launch the prevention partnership, there were a lot of, sort of knee-jerk reactions to ‘Oh where's the hard evidence around prevention? What can you really say about the evidence around prevention?’ (KI 5)

Informants worried that the community’s lack of internal consensus has created fragmented advocacy messages, with some focused on safe motherhood and pregnancy, and others focused on newborn survival (KI7, KI8-10, KI13, KI15-16). Informants urged that there should be one consistent, high-impact advocacy message (KI7, KI10, KI15). (See Box 1 for advocacy suggestions)

Box 1. **Recommended advocacy strategies to increase political recognition of preterm birth**

Informants highlighted three advocacy strategies to help political actors recognize the importance of preterm birth at the global and national levels.

(1) **Frame preterm birth as a health condition that spans the RMNCH continuum of care** (KI2-3, KI6, KI8, KI10, KI15). Addressing preterm birth is essential to achieving the SDG and ENAP targets. Integrate available preterm birth interventions into maternal and child health programs and leverage this approach in advocacy messaging.

Sometimes it’s better to have an advocate that is focused on one issue, like preterm birth, but I think in terms of the ultimate impact it is a much better strategy overall to highlight [preterm birth] within the broader continuum. (KI 8)

(2) **Leverage evidence about the severity of the burden of prematurity as the leading cause of child death** (KI1, KI3, KI5, KI12, KI14-15). Unlike other high-burden conditions, such as HIV/AIDS or malaria, preterm birth is often blended with other factors contributing to newborns deaths (KI10). The importance and impact of investing in preterm birth risks getting lost.

We need to make it simple [for political leaders]. And I would say point one, frame it as a very important problem in terms of burden of disease. (KI12)

(3) **Adopt a universal frame that shows how preterm birth impacts families of all geographies, races, and socioeconomic statuses** (KI1, KI4, KI10-12, KI14). Improve data and messaging on the economic burden of prematurity and articulate the cost-saving potential of preterm birth interventions for policymakers, particularly Ministers of Finance (KI2, KI5, KI9-10, KI12-13) ^24^.

Preterm birth can happen to anybody, and I think that's an important message for politicians and policymakers to hear. So that people understand that it's not just in somebody else's backyard, it's in their own. It's their mothers, their daughters, and their grandchildren that are at risk for preterm birth, just like anywhere else in the world. (KI11)

I think the challenge is that the preterm birth community is not unified, and I don't know if it could be unified, but the differences between the care camp and the prevention camp, I think, impedes our ability to speak with one voice and move forward together as a community. (KI15)

### Political contexts

This study found that the *Born Too Soon* report and ENAP helped create policy windows and provided platforms to advance the visibility of preterm birth. However, there is a need to better leverage the RMNCH agenda and to mobilize political attention to prematurity in LICs and MICs.

The release of the *Born Too Soon* report was an important policy window to bring worldwide attention to prematurity (KI1, KI3-5, KI8-10, KI15) ^3, 7^. To mark the report’s launch, more than 30 organizations made new or enhanced commitments in support of the UN Secretary General’s Every Woman Every Child initiative ^25^. A dedicated launch disseminated the report’s findings to political leaders and received significant media coverage (KI2-4, KI15) ^26^. Later that year, more than 50 countries recognized World Prematurity Day 2012 and several governments made commitments to reduce preterm mortality ^25, 27^.

While *Born Too Soon* brought attention to preterm birth as a single condition, ENAP helped advance and maintain attention to the issue as part of a larger newborn survival agenda (KI2-4, KI7-10, KI15). In 2014, researchers working within ENAP published the Every Newborn series in the *Lancet*, which found that efforts to reduce preventable deaths among newborns have been slow, in spite of existing solutions ^28^. At the 67 ^th^ World Health Assembly (WHA) in May 2014, evidence from the *Lancet* series was highlighted and Melinda Gates gave a keynote address urging action on newborn health (KI10-11, KI15) ^29^. The WHA passed Resolution 67.10, with 194 member states endorsing the ENAP ^30^. As of 2017, 48 countries have established national newborn survival plans or given newborns a platform in national health plans ^31^.

Informants explained that the preterm birth community has struggled to build strong linkages with the RMNCH community and leverage the larger RMNCH agenda for increased political support (KI2-3, KI5, KI7-9, KI12). For example, informants reported that the wider community largely failed to respond when data in 2014 showed preterm birth as the leading cause of death among children under five years of age – the first time an infectious disease was not the leading cause of child mortality (KI8, KI10, KI14-15, KI18) ^32^. Informants described the United Nations International Children’s Emergency Fund (UNICEF) as historically weak on issues of prematurity and newborn survival (KI3, KI6, KI8, KI10, KI12, KI15). In addition, collaboration between the maternal and newborn survival communities has historically been challenging, largely due to competition among RMNCH institutions for limited resources (KI2, KI5, KI7-8, KI10, KI12) ^17^. Informants warned that this fragmentation presents significant barriers to collective action impacting the infant mortality agenda (KI5, KI7, KI10, KI17) ^6^. There are some signs of improvement (KI5). For instance, the 2015 Global Maternal Newborn Health Conference focused on the integration of maternal and newborn health along the continuum of care, and included a session on management of preterm birth and care of the preterm newborn ^33– 35^.

Efforts are also underway to raise political attention to prematurity in LICs and MICs. Informants noted that World Prematurity Day advocacy efforts have been slow, even stagnant, in some high-burden settings (KI2-4, KI14, KI15). Also, newborn health groups such as ENAP and the USAID-led Every-Preemie-SCALE consortium are partnering with high-burden countries to develop country-specific plans to reduce preterm birth rates and improve survival outcomes ^36^. Informants also noted that the GFF might have influence over whether LIC and MIC decision-makers prioritize newborn health at the country-level (KI5, KI10).

The bad news is I don’t believe there’s anyone in these countries, at the moment, when they’re writing their plans, actually in the room, saying “preterm birth, preterm birth.” (KI10)

### Issue characteristics

Data weaknesses present barriers to how well prematurity is understood and how aware policymakers are about the severity of preterm birth. These weaknesses include poor quality of country-level data and gaps in the evidence needed to guide implementation.

Informants highlighted limitations with existing data on the global burden of prematurity (KI1-3, KI7, KI9-14, KI16-17), noting the very wide variability of country-level epidemiological data (KI1-6, KI9, KI12, KI14-17). Data quality varies in part due to different methods of recording gestational age and misclassification of live-born newborn babies as stillbirths ^3^. There was some progress as of May 2019: of the 90 countries that adopted the Every Newborn tracking tool, 41% adopted birth registration and 53% had a perinatal death review policy ^37^. Informants noted a need for more granular data on preterm birth among sub-populations in high-burden settings, in order to understand the effectiveness of interventions and build strategies for prevention (KI4, KI10).

Informants also reported a lack of evidence to guide implementation of effective interventions and ways to address barriers to delivery in different local contexts, highlighting two interventions: (1) administering ACS to women at high risk of premature delivery; and (2) kangaroo mother care (KMC), sometimes called skin to skin contact, to care for preterm infants (KI1-11, KI12, KI14-15). As of 2015, coverage of ACS and KMC was below 10% in LICs and MICs ^20^.

Informants explained that scale-up of ACS in low-resource settings significantly slowed after a 2015 cluster randomized trial found that administering ACS at all levels of care in rural and semi-urban areas led to a population level increase in neonatal deaths ^38^. In light of these findings, at least three organizations stopped or slowed their scale-up of ACS and some researchers called for a complete halt on ACS implementation in low-resource settings until the WHO released new guidelines (KI2-3, KI5-6, K9, KI12-15) ^39^. In 2017, the WHO began a three-year trial on ACS in low-resource settings ^40^. Further, informants underscored the importance of understanding the interaction of local contexts with the planned intervention to ensure that it does not have unintended fatal consequences (KI3-4, KI9, KI12) ^38– 40^.

Despite consensus regarding the effectiveness of KMC in improving premature infant outcomes – including a Cochrane Review and WHO endorsement – informants described debate about the clinical definition of KMC and how best to implement KMC in all settings (KI3, KI5, KI9, KI13-15). Informants explained that a gap in understanding how KMC works in local contexts slows progress to adapt and bring those interventions to scale (KI4-5, KI9, KI11). The WHO has initiated several studies to improve evidence on KMC implementation ^41– 43^.

We are scientifically in agreement that A, B, and C work under a set of ideal circumstances, but those are not immediately applicable and transferable, implementable, in settings where you do not have that set of conditions.
  (KI12)

Nearly half of informants felt that policymakers do not yet recognize the severity of prematurity (KI3, KI8-10, KI12, KI14-15, KI17-18). Despite data challenges, most informants reported that available estimates on the burden and incidence of preterm birth can be used to engage policymakers at the global level and in LICs and MICs (KI1-8, KI12-15). For example, after data showed Malawi with the highest rate of prematurity, the Malawi government launched a national newborn action plan with a strong focus on scaling up KMC and other methods of care for preterm infants (KI15) ^44^.

We know enough to act. It’s not like lack of data should paralyze us or lead to inaction. (KI14)

## Discussion

Our study finds that preterm birth has struggled to achieve political priority on the global health agenda. Since 2012, the preterm birth community has made important progress in developing a well-networked group of stakeholders committed to newborn health, developing groundbreaking evidence on the severity of preterm birth, and supporting the development of country-specific Every Newborn action plans. However, several challenges have hindered global action on preterm birth and threatened much needed progress in the SDG era. These are:

- Limited evidence and a lack of country-level data on the causes of and solutions to prematurity, including prevention strategies and effective implementation in low-resource settings.
- Lack of consensus on how to allocate limited resources across prevention and treatment interventions.
- Difficulties integrating prematurity into the RMNCH agenda and uniting the RMNCH community to leverage support for prematurity.

These challenges echo 2010 findings from Sather and colleagues ^45^. Although there has been some progress, our study finds that many of the same challenges persist and create barriers in attracting and maintaining policymaker attention to preterm birth. For instance, gaps in evidence and locally relevant data can weaken trust in expert recommendations. Conflicting or confusing guidance on interventions makes it difficult for policymakers to determine how they should invest their limited resources. Additionally, it is not clear to policymakers how focusing on preterm birth can drive improvements across RMNCH indicators.

The findings of our study point to four main policy options for the global health community to consider in order to advance the priority of preterm birth moving forward: (1) address data and evidence gaps that hamper implementation; (2) clarify the viable solutions to prevent and address preterm birth; (3) invest in strategies to address preterm birth across RMNCH; and (4) develop coordinated strategies to bring visibility to prematurity within the RMNCH agenda.

### Develop a research agenda to address data and evidence gaps

Informants described a number of data and evidence gaps in understanding the causes, solutions, and effective implementation of interventions in low resource settings. Previous studies have also found substantial gaps in understanding preterm birth ^22, 46^. Although the substantial data gaps are known, priorities for the research agenda are not clear – a 2016 study found that while preterm birth prevention research is likely to have a high impact, it ranked only as 129 ^th^ among 205 priority research questions for newborn survival ^47^. The preterm birth community must develop a coordinated research agenda to help facilitate funding and action for research and data improvements. This could in turn help identify opportunities to leverage other data investments for preterm birth, such as efforts to strengthen national health information systems.

### Clarify viable solutions

Informants described a lack of agreement on strategies to tackle preterm birth, often reflecting false dichotomies, e.g., prevention vs. treatment focus, safe motherhood vs. neonatal health. The preterm birth community must clarify strategies along the continuum of care and develop coordinated recommendations on priority investments, based on robust evidence vetted by the field. Attempting to prioritize preterm birth without clear and viable solutions will discourage policymakers and make it more difficult to hold leaders accountable.

### Invest in preterm birth across RMNCH strategies

Many of the struggles in the preterm birth community can be attributed to limited resources. The preterm birth field faces steep competition for limited resources, from within the RMNCH community and also global health broadly. Internal tension and a lack of consensus within the preterm birth field places the agenda at an even greater disadvantage, because the field lacks a deep network of researchers, policymakers, and other champions when compared with other high-burden health issues that have already been established as clear policy priorities. It is likely that improved evidence and consensus will identify effective preterm birth strategies across the RMNCH continuum of care, which will require increased investment by global and national policymakers. The RMNCH field should identify integrated strategies to sufficiently resource preterm birth prevention and treatment, rather than resorting to former silos and competition for resources that will only hinder progress.

### Increase visibility to prematurity within the RMNCH agenda

The preterm birth community made a strategic decision to broaden its focus to newborn health under the ENAP platform. This pragmatic approach is aligned with the emphasis in the SDGs for an integrated, multi-sector, and multi-stakeholder approach.

However, framing preterm birth as one newborn health condition among many has diluted policymaker focus and stalled action on preterm birth. There is a real risk that preterm birth will continue to be overlooked, jeopardizing progress across the RMNCH continuum.

The challenge is to leverage the RMNCH platform without hindering efforts to reduce preterm birth. The preterm birth community and partners should recognize preterm birth as essential to achieving RMNCH goals in global and national strategic documents and include specific indicators to reduce preterm birth. The RMNCH community should join in coordinated advocacy efforts and help raise the political priority of preterm birth.

### Strengths and weaknesses of the study

To the best of our knowledge, this is the first study of the challenges in assuring global political priority of preterm birth. One major strength is that we interviewed a broad range of stakeholders across multiple types of organizations: researchers, practitioners, advocates, and policymakers at the global level and in LICs and MICs.

There are at least four limitations. First, the study did not examine whether the issue of prematurity is or is not a national level political priority, which could be useful, particularly in high burden countries. As mentioned, we deliberately focused our study at the global level, given the worldwide nature of preterm birth, but it will be helpful to conduct future studies at the national level. Second, our study relied upon self-reporting by key informants, who may not have disclosed full and complete information during our interviews and who may have carried personal biases on key issues. We addressed the potential for bias by granting anonymity to study participants, and sought comments from representatives of different organizations. Third, many of our study participants have collaborated on past projects, which is common in global health but could potentially lead to a lack of diverse perspectives. To address these, we probed in our interviews to uncover and understand areas of difference and consensus among respondents.

Lastly, our study interviews were conducted in 2015. However, we have reviewed and incorporated current reports and updated literature through the year 2019 to capture the most important policy events and publications related to preterm birth from 2015–2019.

## Conclusion

The SDGs targets to end preventable newborn and childhood deaths by 2030 will go unmet if deaths from prematurity continue to climb year after year. To reduce prematurity, global policy makers and national leaders must recognize the problem of preterm birth as a priority issue.

This study identified actionable ways the preterm birth and newborn health community can increase the political priority of preterm birth on the global health agenda: develop a research agenda to address data and evidence gaps, clarify viable solutions, invest in preterm birth across RMNCH strategies, and elevate the visibility of preterm birth as an important issue within the RMNCH continuum. These actions will help enable greater political priority to preterm birth, which has the potential to spur global and country-level advancements to reduce prematurity and achieve SDG targets.

## Data availability

### Underlying data

We are unable to share the underlying data due to data protection issues. Interview transcripts are the primary source of data for this manuscript. Data cannot be shared publicly because to do so would be a breach of confidentiality. The participants in the key informant interviews could be identified using the transcripts, which is why the data cannot be made available.

The University of California San Francisco (UCSF) Committee on Human Research certified this study, number 15–15752, as exempt.

Readers with questions about the data can contact the corresponding author by email at sara.kassabian@gmail.com. If there is interest in secondary analyses of the data, we will review the request and consider purpose, methods, and use of the data.

Associated materials (semi-structured interview guide, codebook) have also been made available at the UK Data Repository.

### Extended data

The semi-structured interview guide and codebook are included in the UK DataService ReShare repository.

Repository: Building a global policy agenda to prioritize preterm birth: A qualitative analysis on factors shaping global health policy-making Colchester, Essex: UK Data Service. https://dx.doi.org/10.5255/UKDA-SN-854251 ^13^.

This project contains the following underlying data:

- **Semi-structured interview guide**. (The semi-structured interview guide includes questions that probe the key domains that underlie the Shiffman and Smith Framework for Political Priority: Actor Power, Ideas, Political Contexts, and Issue Characteristics.)
- **Codebook**. (The codebook was created to define the meanings behind different codes.)

Data are available under the terms of the Creative Commons Zero "No rights reserved" data waiver (CC0 1.0 Public domain dedication).
