# Supplementary material for: Building a global policy agenda to prioritize preterm birth: A qualitative analysis on factors shaping global health policymaking
Source: Gates Open Res. 2020 Jun 22;4:65. [Version 1] doi: 10.12688/gatesopenres.13098.1 (PMC7578407; doi:10.12688/gatesopenres.13098.1)
Supplement: Peer review history

# Peer review: Reviewer response for version 1

**DOI:** 10.21956/gatesopenres.14257.r29672

**Published:** 2020-10-20

**Authors:** Kristine Husøy Onarheim

**Affiliations:** 1 Institute for Global Health, University College London, London, UK

This important paper aims to identify factors that shape the global political priority of preterm birth. It is a well-written and timely paper, and provides new insights to why preterm birth has not received policy priority despite its large burden. These findings are highly relevant for the prematurity and child mortality community, but also in our understanding of agenda setting in global health more generally. The paper helps explain why prematurity is not higher on the global health agenda. Further, the paper highlights relevant suggestions on how to increase its priority. While these contributions are important, I have some concerns with the paper in its current presentation, and in particular the description of the review.

**Methods:**

- **Review:** The review of the literature is informative to understanding the policy context and developments after the interviews were conducted. However, the methods used are unclear and the choice of sources seem to miss out on potentially relevant literature. I would recommend addressing these, or if not, being explicit about the limitations of the review:
   
  - While the academic literature is likely to cover some of the changes in policy priority (2015-2019), I am not convinced that PubMed and Google scholar literature will be up to date on policy development. Given that policy making also is influenced by other developments than new evidence, I wonder if this is captured when the search focused on academic databases. Why were policy documents not included in your review? I think this would have added value, with attention to how prematurity was (or was not) on the agenda in WHO documents, guidelines, etc. This would also align with your attention on the SDG era and provide insights on (other) issues that are prioritized. This could help you explain the hindrances for why prematurity is not high on the agenda.
  - The search strategy conducted is not described in detail, and it is hence difficult for the reader to evaluate whether the search captured relevant elements. While I understand that the manuscript did not aim to conduct a systematic review, a detailed presentation of your scoping review should be included. I would appreciate if the search strategy could be added as a supplement/appendix. The presentation of the review of the manuscript seems more as tool to inform the findings from your qualitative study than a review, and it is not clear in the Results section where you draw upon your findings from the review. I would suggest to either provide more detail about how the review was conducted and demonstrate clearer in the Results section how it informed your study, or to leave out the review part and consider your ‘coping review’ as a way to understand your findings. The key strength of your paper is the qualitative interviews and the rigorous analysis.
- **Political priority framework:** This study adapts the Shiffman and Smith framework, which is a well-chosen framework for this study. The authors write that the framework has been applied to ‘underrepresentative’ health challenges. This normative statement about the framework highlights the relevance of the framework to also understand why certain issues are not on the agenda, but I argue that it is equally useful to understand why some issues do get attention. I would suggest to rephrase (or something similar): ‘The framework has been applied to understand the factors contributing to or hindering policy action in global health, including safe motherhood, newborn survival, mental health, and surgical care’.
- **Interviews and recruitment:** Well-described methods used to recruit study participants. The information about using a combination of purposive and snowball sampling could have been presented in abstract.

 

**Results:**

- **Issue Characteristics: **The last paragraph describes that half of the informants say that prematurity is not perceived as a severe problem. Were they able to say something about why? It seems like there was detailed discussion in specific parts of the prematurity agenda (e.g. KMC), but what is the issue not perceived as severe? This links to your findings on ideas (and external framing). It would be of great interest to know if they said more that could explain why this is not perceived as an severe issue (by some).
- The authors also highlight when their claims are supported by multiple (or some) stakeholders, and show that insights are gained from the broader sample. I would suggest to keep the information about who said what.

 

**Discussion:**

- The discussion is well-written and reflects upon the main findings of the study. I have some questions concerning the interpretation of the study findings:
   
  - On your finding on *Integration difficulties* and recommendation to 4 on coordination of strategies to make prematurity visible on the RMNC agenda: How is lack of priority to prematurity a) different from and/or b) similar to attention to newborn health and maternal mortality (which both are higher on the agenda, in particular maternal health)? As your paper (and stakeholders) argue, there are reasons to believe that there is an overlap in interventions in this era, but why has there been less attention to prematurity than what has been seen in the increased attention for maternal survival (Smith and Shiffman, 2016 ^1^. Your study describes findings for prematurity, but the discussion would benefit from reflecting on these in relation to the existing literature on issue attention in newborn health, maternal health and stillborns (the Lancet series give some insights in why stillborn is low on the agenda)?
  - *Increase visibility to prematurity:* You discuss the fine balance between the risks of prematurity becoming less visible and potential benefits if prematurity is incorporated (and/or overlooked) in the broader RMNCH agenda. Can you – based on your interview/findings – say anything about how causes (prematurity) might be seen as different than newborns, children, mothers (individuals) that often are core in the messaging on the RMNCH agenda? Is the framing of prematurity as a particular problem (and cause of child deaths) found useful outside the prematurity community? It seems as the collective works well on the inside, but less in its external engagement, which may influence visibility – in particular when there are few low-hanging fruits and solutions. I might be wrong, but it would be helpful if you can reflect upon some of this in more detail in the Discussion section.

 

 Overall, the paper is well-written and of great interest for global health scholars. With the exception of the lack of clarity on review (see comment above), the study design and analysis is rigorous. I hope comments are helpful to improve the manuscript further.

Is the work clearly and accurately presented and does it cite the current literature?

Yes

If applicable, is the statistical analysis and its interpretation appropriate?

Not applicable

Are all the source data underlying the results available to ensure full reproducibility?

No

Is the study design appropriate and is the work technically sound?

Partly

Are the conclusions drawn adequately supported by the results?

Yes

Are sufficient details of methods and analysis provided to allow replication by others?

Partly

Reviewer Expertise:

Priority setting and policy making in global health (child and newborn health, women's health, migrant health, universal health coverage), including policy analysis, qualitative studies (and quantitative studies).

I confirm that I have read this submission and believe that I have an appropriate level of expertise to confirm that it is of an acceptable scientific standard, however I have significant reservations, as outlined above.

## References

[1] : Setting the global health agenda: The influence of advocates and ideas on political priority for maternal and newborn survival. *Soc Sci Med*.166: 10.1016/j.socscimed.2016.08.013 86-93 10.1016/j.socscimed.2016.08.013 27543685PMC5034850

# Peer review: Reviewer response for version 1

**DOI:** 10.21956/gatesopenres.14257.r29564

**Published:** 2020-10-08

**Authors:** Mohan Paudel

**Affiliations:** 1 Research, Monitoring and Evaluation Department, Adara Development (Australia), Sydney, NSW, Australia; 2 Initiative for Research, Education and Community Health Nepal, Kathmandu, Nepal

Congratulations to the authors for this excellent piece of work. The manuscript is very well led right from abstract to the conclusion. The key messages are clear and very crucial to reach out to global health communities. Relating these findings to my own work experience of MNCH in Nepal and Uganda, I am in complete agreement with the key challenges around lack of evidence on and country level data, lack of consensus on use of the limited resources and difficulties in integration of prematurity into RMNCH agenda. The policy options outlined by the authors, particularly to address evidence gaps and clarify the viable solutions to prevent preterm birth also resonates very well with my own experience. Policy call to improve data quality on preterm birth at national level, and investment priority to generate evidence on what causes preterm birth are the most important messages from the manuscript.   

 Regarding the methodological detail, the authors have very well applied Shiffman and Smith framework and adapted this to guide them to interview KIs and the analysis. I have some small comments, which I think would help further to improve the quality of the paper:

- In data collection and analysis, the authors said interviews were conducted until the study reached theoretical saturation, i.e. no new themes *emerged* in the interviews. From the result sections, readers note that actor power, ideas, political contexts and issue characteristics are the themes presented in the paper, which indeed *did not emerge, rather were directed* *by the framework *the authors used.
- Interview transcripts were *analyzed* using Dedoose, version 7.1.3, maybe the authors want to amend the language. Dedoose facilitated coding process, analysis is more about the framework the study was guided by and the actual coding process used.
- It would help readers if the authors write a few lines about ‘impression coding’ as they use this term in the analysis section.
- Each interview was coded by two researchers SK and SF to ensure consistency. As the authors were informed by a very clear Shiffman and Smith framework to direct their analysis, I suppose there is a very less chance of inconsistency among the coders. It might help readers to elaborate a few lines on what were the inconsistencies like if any.
- In the strengths and weakness section, the authors said they relied on ‘self-reporting by key informants’. It is less clear whether it is a strength or weakness as authors said they used purposively selected KIs as the study participants. The authors might want to check the methodology section for consistency.

Overall, the paper is excellently written, and very worthy to go out for indexing.

Is the work clearly and accurately presented and does it cite the current literature?

Yes

If applicable, is the statistical analysis and its interpretation appropriate?

Not applicable

Are all the source data underlying the results available to ensure full reproducibility?

No source data required

Is the study design appropriate and is the work technically sound?

Yes

Are the conclusions drawn adequately supported by the results?

Yes

Are sufficient details of methods and analysis provided to allow replication by others?

Yes

Reviewer Expertise:

MNCH, HIV/AIDS, Primary Health Care, Health Systems and Policies

I confirm that I have read this submission and believe that I have an appropriate level of expertise to confirm that it is of an acceptable scientific standard.

# Peer review: Reviewer response for version 1

**DOI:** 10.21956/gatesopenres.14257.r29344

**Published:** 2020-10-01

**Authors:** Abigail Kazembe

**Affiliations:** 1 Kamuzu College of Nursing, University of Malawi, Zomba, Malawi

This is a well written article, which has highlighted preterm issues at policy level and the report will contribute towards improvements of preterm neonates. The framework used is appropriate and the researchers need to be commended. The method for data collection is also appropriate, however presentation of results lack information about the sample. At least organizations that took part without necessarily describing their post should have been communicated. We depend on the same institutions to push the preterm agenda, as such it is important to hear their views specifically.

 The authors acknowledge that the study was conducted some years back and are hoping that the literature review will support the findings. There is need to indicate dates for the documents used to ensure that we are indeed using current data. What criteria was used to select these documents? How many did they find? How many were discarded and why were they discarded? It is important to describe the process.

 Once these comments are addressed the article could be indexed.

Is the work clearly and accurately presented and does it cite the current literature?

Yes

If applicable, is the statistical analysis and its interpretation appropriate?

Not applicable

Are all the source data underlying the results available to ensure full reproducibility?

Partly

Is the study design appropriate and is the work technically sound?

Yes

Are the conclusions drawn adequately supported by the results?

Yes

Are sufficient details of methods and analysis provided to allow replication by others?

Partly

Reviewer Expertise:

NA

I confirm that I have read this submission and believe that I have an appropriate level of expertise to confirm that it is of an acceptable scientific standard, however I have significant reservations, as outlined above.

# Peer review: Reviewer response for version 1

**DOI:** 10.21956/gatesopenres.14257.r28970

**Published:** 2020-07-06

**Authors:** Marie A. Brault

**Affiliations:** 1 Department of Social and Behavioral Sciences, Yale School of Public Health, New Haven, CT, USA

I enjoyed reviewing this article discussing the factors that shape leadership and political prioritization of preterm birth. The use of the Shiffman and Smith framework is very successful in this manuscript, as assessing issues like leadership and advocacy can be challenging. The manuscript also provides several helpful recommendations and areas for future research which will be critical for addressing the gaps or challenges identified by the key informants. I have a few specific comments and questions, which would strengthen the manuscript.

- The authors state that the interview data was supplemented with a literature review. Did the authors consider doing this as a systematic review, and/or are there previously published systematic reviews on preterm birth policy or advocacy? Was any data abstraction tool developed to abstract information from the literature (particularly the grey literature)?
- The choice to cite statements in the results with specific key informant IDs is a little unusual and distracting. Unless there is a specific reason to do so, I would encourage the authors to remove these citations.
- The ID numbers for the quotes are meaningless on their own. I understand that the authors want to indicate that different quotes came from different participants, while also maintaining confidentiality. However, it would be helpful to add a little more information on the participants, perhaps a general description of their role/profession (clinician, epidemiologist, basic science research, etc.) and perhaps the number of years they have been working in the area.
- On page 7, please define the acronym ACS.
- Did key informants or the literature describe any LMICs that were doing better than their peers at leadership and advocacy for preterm birth? Given the many implementation challenges identified, it would be helpful and critical to also discuss those countries or settings that have made progress. Malawi is identified as a country that has been responsive to making changes based on available data, but little information is provided on how they were able to accomplish that.
- The authors note that one limitation of the qualitative data is that it was collected in 2015, but that the literature review allowed the data to be brought more up to date. However, it was not clear how the literature review was used to supplement the interviews. Specifically, is there more current evidence on the use of antenatal corticosteroids and/or kangaroo care? It would be helpful to be a little more explicit on whether the literature indicates changes or movement on preterm birth since the interviews were conducted.
- Adequate and comparable data from LMICs is a challenge for child health beyond the perinatal and neonatal period (see Brault *et al.*, 2020 ^1^ for a discussion of this). Given limited resources and the many competing demands for reporting that health workers face, what specific recommendations did the key informants (or authors) have on how data collection efforts can be improved?

Is the work clearly and accurately presented and does it cite the current literature?

Partly

If applicable, is the statistical analysis and its interpretation appropriate?

Not applicable

Are all the source data underlying the results available to ensure full reproducibility?

No

Is the study design appropriate and is the work technically sound?

Yes

Are the conclusions drawn adequately supported by the results?

Yes

Are sufficient details of methods and analysis provided to allow replication by others?

Partly

Reviewer Expertise:

I am a medical anthropologist with expertise in qualitative and mixed methods approaches, and maternal, neonatal, child, and adolescent health both in the U.S. and internationally.

I confirm that I have read this submission and believe that I have an appropriate level of expertise to confirm that it is of an acceptable scientific standard, however I have significant reservations, as outlined above.

## References

[1] : Measuring child survival for the Millennium Development Goals in Africa: what have we learned and what more is needed to evaluate the Sustainable Development Goals?. *Glob Health Action*.2020;13(1) : 10.1080/16549716.2020.1732668 1732668 10.1080/16549716.2020.1732668 32114967PMC7067162
